# Genome Analysis of *Pseudomonas aeruginosa* Strains from Chronically Infected Patients with High Levels of Persister Formation

**DOI:** 10.3390/pathogens12030426

**Published:** 2023-03-08

**Authors:** Amr A. Baiomy, Fathy E. Serry, Ashraf A. Kadry, Galal Yahya, Swapnil Doijad, Ahmed Mostafa, Mobarak Abu Mraheil, Amira M. El-Ganiny

**Affiliations:** 1 Microbiology and Immunology Department, Faculty of Pharmacy, Zagazig University, Zagazig 44519, Egypt; amrelhalawaty@gmail.com (A.A.B.); fathyserry@hotmail.com (F.E.S.); kadry57@yahoo.com (A.A.K.); galalmetwally2020@gmail.com (G.Y.); 2 Institute of Medical Microbiology, German Center for Infection Giessen-Marburg-Langen, Justus Liebig University Giessen, Schubertstrasse 81, 35392 Giessen, Germany; swapnil.doijad@gmail.com; 3 Water Pollution Research Department, Environment and Climate Change Research Institute, National Research Centre, Giza 12622, Egypt; ahmed_elsayed@daad-alumni.de; 4 Center of Scientific Excellence for Influenza Viruses, National Research Centre, Giza 12622, Egypt

**Keywords:** persister cells, *Pseudomonas aeruginosa*, multidrug-tolerant cells, killing curve, whole genome sequencing (WGS), resistome profiling, phylogenetic analysis

## Abstract

The appearance of persister cells with low metabolic rates are key factors leading to antibiotic treatment failure. Such persisters are multidrug tolerant and play a key role in the recalcitrance of biofilm-based chronic infections. Here, we present the genomic analyses of three distinct *Pseudomonas aeruginosa* Egyptian persister-isolates recovered from chronic human infections. To calculate the persister frequencies, viable counts were determined before and after treatment with levofloxacin. The susceptibilities of isolates to different antibiotics were determined using the agar-dilution method. To determine their recalcitrance, the levofloxacin persisters were further challenged with lethal concentrations of meropenem, tobramycin, or colistin. Furthermore, the biofilm formation of the persister strains was estimated phenotypically, and they were reported to be strong biofilm-forming strains. The genotypic characterization of the persisters was performed using whole genome sequencing (WGS) followed by phylogenetic analysis and resistome profiling. Interestingly, out of the thirty-eight clinical isolates, three isolates (8%) demonstrated a persister phenotype. The three levofloxacin-persister isolates were tested for their susceptibility to selected antibiotics; all of the tested isolates were multidrug resistant (MDR). Additionally, the *P. aeruginosa* persisters were capable of surviving over 24 h and were not eradicated after exposure to 100X-MIC of levofloxacin. WGS for the three persisters revealed a smaller genome size compared to PAO1-genome. Resistome profiling indicated the presence of a broad collection of antibiotic-resistance genes, including genes encoding for antibiotic-modifying enzymes and efflux pump. Phylogenetic analysis indicated that the persister isolates belong to a distinct clade rather than the deposited *P. aeruginosa* strains in the GenBank. Conclusively, the persister isolates in our study are MDR and form a highly strong biofilm. WGS revealed a smaller genome that belongs to a distinct clade.

## 1. Introduction

*Pseudomonas aeruginosa* is an opportunistic pathogen that poses a threat in clinical settings due to its intrinsic and acquired resistance to a wide range of antibiotics and biocides [1,2,3]. During chronic infection, *P. aeruginosa* can be evolved toward a high-persister state, producing more antibiotic-tolerant cells [4]. Persister-cell formation is a phenomenon that contributes to the tolerance of a bacterial sub-population to antimicrobial agents. The presence of persister cells surviving high concentrations of antibiotics makes it virtually impossible to eradicate the chronic infection caused by *P. aeruginosa*. Although persisters constitute a small fraction of the population, they can be selected by surviving the treatment with high doses of bactericidal antibiotics [5]. Notably, this antibiotic tolerance of persister cells is distinct from genetically inherited resistance [6].

Although persister cells were discovered in *Staphylococcus* sp. as early as 1944 by Bigger [7], they were ignored for a long time. The first mutants identified to be altered in their persistence were the high persistence (*hip*) mutants in *Escherichia coli* that were discovered in 1983 [8]. Furthermore, persister cells have been described for other pathogens, including *P. aeruginosa, Mycobacterium tuberculosis, Salmonella* sp., and *Streptococcus* sp. [6,9]. The existence of persisters is believed to prolong and exacerbate the treatment of infectious diseases, such as tuberculosis and cystic fibrosis (CF)-associated lung infections [10].

Biofilm formation by bacteria is highly associated with increased resistance to conventional antibiotic therapy. *Pseudomonas* possesses a strong tendency to develop biofilm, which aids in its persistence and contributes to recalcitrant and/or recurrent infections after antibiotic therapy [11,12,13]. The biofilm growth mode creates micro-environment conditions that activate stringent response mechanisms and toxin–antitoxin (TA) systems that render the bacterial population dormant and hence highly tolerant to antibiotics [14].

Persister cells represent a stage of dormancy that protects them from complete eradication by antimicrobial substances, even in the presence of concentrations that vastly exceed the minimal inhibitory concentration (MIC). Persister cells are genetically identical to antibiotic-sensitive bacteria within a population but have a distinct phenotype in that they are tolerant to antibiotics [15,16]. The dormancy stage in persister cells is thought to be the underlying mechanism of antibiotic tolerance, since most antibiotics target bacterial components or pathways involved in replication [17]. Nevertheless, persister cells can switch from the dormant to the replicating stage. This ‘bet-hedging’ strategy is thought to be a survival strategy of microbial populations [18]. 

It has been reported that the level of persisters could be increased as a result of a heritable mutation in genes such as *hipA* (encodes the toxin entity of the toxin-antitoxin module *hipBA*) [8]. After 30 years of reporting *hipA* mutants, it was found that HipA inactivates glutamyl tRNA synthetase (GltX) by phosphorylation [19]. The inhibition of GltX stimulated the synthesis of guanosine pentaphosphate/tetraphosphate (ppGpp); ppGpp is a very important stress alarm, one that dramatically increases the persistence level [20,21].

Although persister cells can arise from stochastic events in growing cultures [22], evidence suggests that their formation can also be induced as a response to several environmental factors, such as nutrient and oxygen deprivation, oxidative stress, DNA damage, and antibiotics [23]. The addition of levofloxacin at a concentration of 10–100 times higher than its specific MIC was capable of inducing and separating persister cells [24]. Starvation, hypoxia, and antibiotic stress could be directly correlated to a reduced metabolic rate and decreased cellular activity. Reduced metabolic activity is related to increased persistence [25]. 

Previous reports have suggested that persister cells exhibit different phenotypes compared to the wild-type strains, including smaller colony sizes or changes in pigmentation [10,14,26]. Given that persister cells can enter a non-growing state, tolerate high concentrations of bactericidal antibiotics (for example by activating efflux pumps), and regrow once the treatment is ceased, these cells have already been linked to the relapse and recalcitrance of chronic infections [14]. 

This study aimed to examine the fraction of persister cells in the *P. aeruginosa* cultures of clinical isolates and to determine the susceptibility to different antimicrobials and the biofilm-forming capacity for *P. aeruginosa* persister isolates as compared to the reference strain PAO1. Moreover, whole genome sequencing, resistome profiling, and phylogenic analysis were performed for the three persister isolates compared to the first sequenced *P. aeruginosa* strain (PAO1 genome). 

## 2. Materials and Methods

### 2.1. Bacterial Strains and Ethical Statement

Thirty-eight *P. aeruginosa* isolates were obtained from different sources (urine, sputum, and burn specimens) from patients admitted to El-Ahrar Educational Hospital and Zagazig University Hospital, Egypt. The standard strain of *P. aeruginosa* PAO1 was included in this study. It was provided from the stock culture collection of the Microbiology and Immunology Department, Faculty of Pharmacy, Zagazig University. 

Experiments involving human samples were performed in accordance with the Declaration of Helsinki and were approved by the Zagazig University Institutional Review Board (Approval number: ZU-IRB-10360).

### 2.2. Antimicrobial Agents

The antibiotics used in the study were pharmaceutical grade products. Meropenem was obtained from the ADWIA Company, Egypt. Cefoperazone and tobramycin were obtained from the Egyptian Pharmaceutical Industries Company (EPICO), 10th of Ramadan city, Egypt. Cefepime was obtained from RAMEDA, Egypt. Levofloxacin and amikacin were obtained from the AMOUN Company, Egypt. Colistin and chloramphenicol were the products of SIGMA-TEC, 6th October City, Egypt. The batch numbers of the used antimicrobials were listed in Supplementary Table S1.

### 2.3. Determination of Viable Bacterial Count 

Ten-fold serial dilutions (final volume 1000 μL) of the bacterial suspensions, from an overnight culture in a nutrient broth (Oxoid, Hampshire, England), were performed in Eppendorf tubes. Triplicates of 100 μL of the appropriate dilutions were plated on nutrient agar plates (Oxoid, Hampshire, England). The plates were incubated for 24–48 h at 37 °C. The average numbers of colonies on the appropriate plate were counted and the colony forming units (CFU)/mL were calculated [27].

### 2.4. Selection and Quantification of Levofloxacin Persister Cells 

Levofloxacin was added to a growing nutrient broth culture of the isolate at a concentration of 10–100 times its MIC. The suspensions were incubated in a shaker incubator (200 rpm) at 37 °C for 24 h. Falcone tubes containing bacterial cultures were centrifuged for 5 min at 8000 rpm and the supernatant was removed without disrupting the pellet. The cells were washed by re-suspending them in 1 mL phosphate-buffered saline (PBS) and transferred to an Eppendorf tube for centrifugation for 5 min at 14,000 rpm. The supernatant was gently discarded and the pellets were re-suspended in 1 mL of Mueller–Hinton broth (MHB). The viable count was made as described previously [28].

### 2.5. Determination of the MIC of Tested Antibiotics 

The MICs of antibiotics were determined using the agar dilution method, according to the clinical laboratory standard institute (CLSI) guideline [29]. The used antibiotics were levofloxacin, chloramphenicol, amikacin, cefoperazone, meropenem, tobramycin, cefepime, and colistin. The MICs were reported and interpreted as sensitive, intermediate, or resistant, according to the CLSI breakpoints indicated in Supplementary Table S2.

### 2.6. The Killing Curve of Bactericidal Antibiotics against Levofloxacin Persisters 

Time-killing curves were performed for selected persister-forming strains with antibiotics, as described previously [30]. Three water-soluble antimicrobial agents (colistin, meropenem, and tobramycin) were selected for time-killing curve assay. 

Briefly, the overnight cultures of *P. aeruginosa* cells, treated with 100X MIC of levofloxacin (~2–5 × 10^3^ CFU/mL), were pelleted (at 6000 rpm for 20 min), washed, and resuspended in 1 mL of a double-strength broth. The stock solutions of antimicrobial agents were prepared with concentrations equivalent to their 2x MICs and added to the suspension of persister cells. Tubes were incubated in a shaker incubator (200 rpm) at 37 °C for up to 24 h. Viable counts were made at zero time and after that at 1, 3, 5 and 24 h. The experiments were performed in triplicate with three independent cultures.

### 2.7. Biofilm Formation Capacity Test 

The quantitative assay of biofilm formation was performed according to Stepanovic et al. [31]. A suspension of tryptone soy broth (TSB, Oxoid, Hampshire, UK) was prepared from the overnight culture of each isolate and adjusted to 10^6^ CFU/mL in the freshly prepared culture broth of TSB. Aliquots of 200 µL of the bacterial suspension were transferred to the wells of a flat-bottom microtiter plate and incubated at 37 °C for 48 h. The TSB was removed gently and distilled water was used to wash the plates to remove any planktonic cells with subsequent air drying. After 20 min of treatment with 200 µL of 99% fixing methanol, the biofilm was then stained with 200 µL of 1% crystal violet (CV) solution for 15 min. After washing of the plate, 33% glacial acetic acid was used as a solvent for CV and the optical density (OD) of the solubilized dye was measured at 570 nm with a spectrofluorimeter (Biotek, Winooski, VT, USA). Wells containing only media were included in the study to serve as a negative control. The experiment was performed in triplicate. 

The cut-off optical density (ODc) was calculated as three times the standard deviations above the mean OD of the negative control. The tested strains were classified into: non-biofilm producer (OD ≤ ODc), weak biofilm producer (OD > ODc, but ≤2 × ODc), moderate biofilm producer (OD > 2 × ODc, but ≤ 4 × ODc), and strong biofilm producer (OD > 4 × ODc), as described previously [31].

### 2.8. Genomic DNA (gDNA) Extraction and Whole Genome Sequencing

The GeneJET™ Genomic DNA Purification Kit was used for gDNA extraction. The DNA purification protocol was performed according to the manufacturer’s instructions. The purified gDNA was used immediately or stored at −20 °C. For the whole-genome sequencing, genomic, short-read sequencing was performed on an Illumina NextSeq 500 sequencer (Illumina, The Netherlands) using a library prepared with MiSeq v3 (2 × 75 bp) reagent kit.

### 2.9. Genome Annotation and Phylogeny

The raw reads were filtered for adaptor sequences and low-quality bases using Trimmomatic v0.36 [32] and assembled de novo using Unicycler v0.4.8 [33]. For both tools, the default parameters were used. Only contigs > 500 bp were considered for further analysis. Genome-based annotation was carried out using PROKKA v1.11 [34]. Single nucleotide variants (SNVs) were identified by mapping filtered reads against the closed reference genome of *P. aeruginosa* PAO1 (NC_002516) using Snippy v4.3.6 (https://github.com/tseemann/snippy (accessed on 5 January 2023)). Furthermore, the isolates were identified using TYGC server (https://tygs.dsmz.de/ (accessed on 5 January 2023)), which compared the genomes to type-strain and calculated the *in-silico* DNA–DNA hybridization score [35].

All the available (as of June 2022) completely closed genomes of 445 isolates were downloaded from NCBI using the NCBI-genome-download script (https://github.com/kblin/ncbi-genome-download (accessed on 5 January 2023)). The phylogeny was constructed based on the MASH distance using Ondov et al. [36]. The multilocus sequence typing was carried out using MLST script (https://github.com/tseemann/mlst (accessed on 5 January 2023)). The pangenome atlas was created using gView Server (https://server.gview.ca/ (accessed on 5 January 2023)). 

### 2.10. Resistome Analysis

The gene differences among the isolates were studied by calculating the core and pan-genome using the Panaroo tool v1.2.3 [37]. Virulence gene profiling was performed by aligning the amino-acid sequences of all genes (using 70% query coverage and 80% nucleotide identity) against the ‘virulence factor database (VFDB) using the Diamond v0.8.36.98 tool [38]. Antibiotic resistance gene profiling was carried out using NCBI’s AMRfinder (https://www.ncbi.nlm.nih.gov/pathogens/antimicrobial-resistance/AMRFinder/ (accessed on 8 January 2023)) as well as the ‘resistance gene identifier’ v5.05.5 against the CARD database (v3.0.3) [39]. The circular image was created using the BRIG tool v0.95 [40]. The heatmap for clustering AMR elements was constructed using ClustVis tool [41]. All the bioinformatics tools were run with default parameters, unless specified.

## 3. Results

### 3.1. Separation of P. aeruginosa Persister Cells and Determination of Their Antimicrobial Susceptibility 

Thirty-eight *P. aeruginosa* isolates taken from chronically ill patients were studied for persister cells formation using 100*X* MIC of levofloxacin. From the thirty-eight *P. aeruginosa* isolates, only three isolates (8%) yielded persisters; these three isolates were isolated from respiratory tract infections and from patients with cystic fibrosis and were named P1, P2, and P3. 

The resistance profile of these persisters showed increased resistance patterns to seven tested antimicrobials (Table 1). In comparison to the reference strain PAO1, all persister strains were resistant to meropenem, levofloxacin, chloramphenicol, and tobramycin (100%), while 90% of these strains were resistant to cefoperazone and colistin. Respective resistance was observed against cefepime (60%) and amikacin (70%). The three persister strains were found to be MDR. 

### 3.2. Survival Curve of Persisters in the Presence of Levofloxacin, Tobramycin, Meropenem, and Colistin 

**Figure 1:**
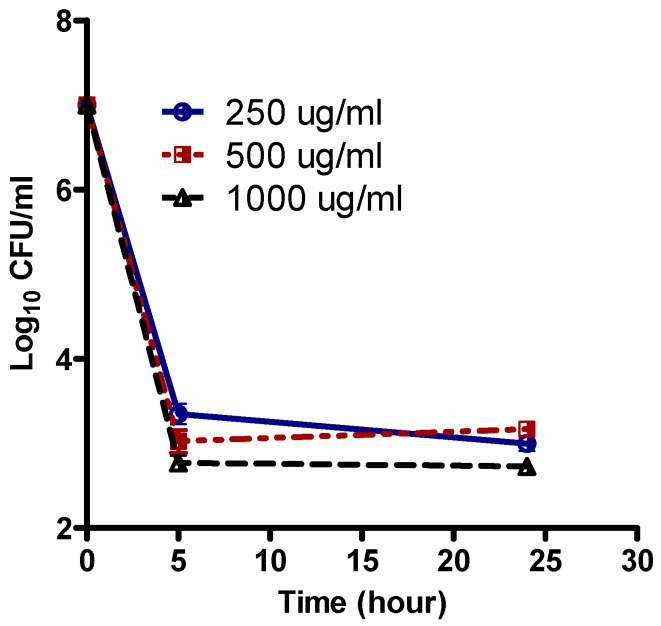
Population analysis curve of levofloxacin against *P. aeruginosa* persister (isolate P1). Levofloxacin was added at time zero at the indicated concentrations to a stationary-phase culture. Data are expressed as the Mean ± SEM of three independent experiments.

After overnight growth in Mueller–Hinton (MH) broth, (Oxoid, Hampshire, England), cells were exposed to increasing concentrations of levofloxacin (250, 500, 1000 μg/mL). The analysis of the number of survivor cells after 5 and 24 h (Figure 1) showed that the bulk of the population was effectively killed with 250 µg/mL levofloxacin. The killing curves were followed on the original strains.

**Figure 2:**
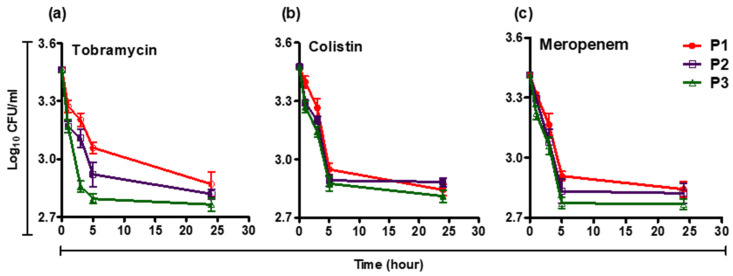
Biphasic time killing curves of (**a**) tobramycin, (**b**) colistin, and (**c**) meropenem against levofloxacin persisters of *P. aeruginosa*. After treatment with 100X-MIC of levofloxacin, *P. aeruginosa* strains (P1, P2, P3) were exposed to bactericidal concentrations of antibiotics and viable counts were determined at different time points (0, 1, 3, 5, and 24 h). Data are expressed as the Mean ± SEM of three independent experiments.

A small fraction of cells (~10^−3^) were tolerant for up to 1000 µg/mL levofloxacin (~100*X* MIC), The persisters after treatment with levofloxacin were exposed to lethal concentrations of colistin, meropenem, or tobramycin, and the survivor’s numbers were followed for 24 h (Figure 2).

### 3.3. Biofilm Forming Capacity of Isolates

**Figure 3:**
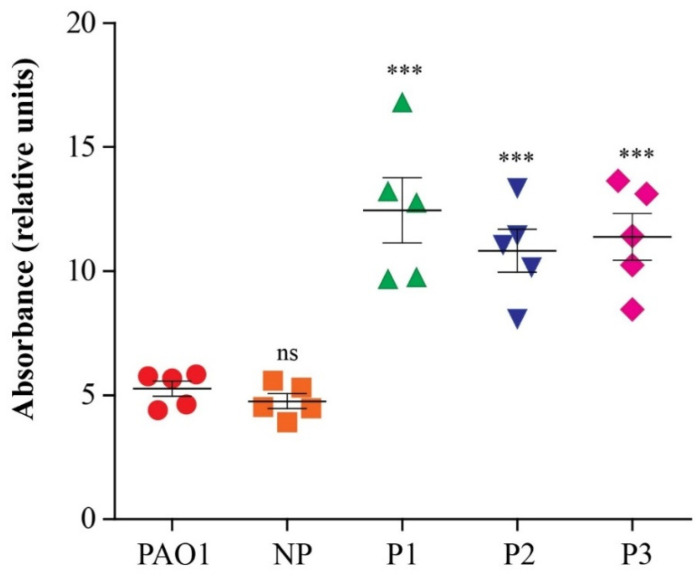
Biofilm formation by persister isolates compared to PAO1 standard strain and a non-persister (NP) clinical isolate. The quantitative assay of biofilm formation was performed using a spectrophotometric assay. All isolates formed strong biofilm with the highest strength observed with persister isolates. Data are expressed as the Mean ± SEM of three independent experiments. Two-tailed unpaired T-tests were employed to analyze significance; ns denotes no significance, *** *p* < 0.001.

The biofilm-forming capacity of the three levofloxacin-persister isolates in addition to the PAO1 strain and a non-persister forming isolate (clinical isolate which had no survival cells after treatment with high concentration (100X MIC) of levofloxacin) was quantified using a spectrophotometric assay. All the tested isolates were strong biofilm producers, as the measured OD was greater than 4ODc (the ODc was 0.1). The persister-forming isolates showed the highest absorbance compared to PAO1, indicating their higher biofilm-forming capacity; the relative absorbance was calculated in comparison to the absorbance of ODc (Table 2 and Figure 3).

### 3.4. Whole Genome Sequencing (WGS) Reveals Distinct Features of the Persisters

All the genome sequences were submitted to the public database under the project accession number PRJNA890046. Genomes of the P1, P2, and P3 isolates were deposited as 1 (SRR21888463), 2 (SRR21888462), and 4 (SRR21888461). Isolates were identified to be *Pseudomonas aeruginosa*, as all the three isolates showed more than 70% DDH to type strain. The genome sizes of the persister isolates were 4,471,320 bp, 4,951,902 bp, and 4,661,420 bp for the isolates P1, P2, and P3, respectively, compared to 6,264,404 bp the genome size of the PAO1_NC_002516 reference strain. The genomes were distinguished into 1547, 1688, and 1706 contigs, respectively. The numbers of protein-coding sequences within the genomes were 4606, 5130, and 4928 genes, for the persister isolates P1, P2, and P3, respectively, compared to the 5671 coding genes for the PAO1 reference strain. The GC contents of the genomes were 64.38, 63.91, and 64.14%, respectively, compared to 66.56% for the PAO1 genome. WGS confirmed species-level identification of all isolates with percent identity values of 98.5% for all assemblies to the reference strain PAO1. 

**Figure 4:**
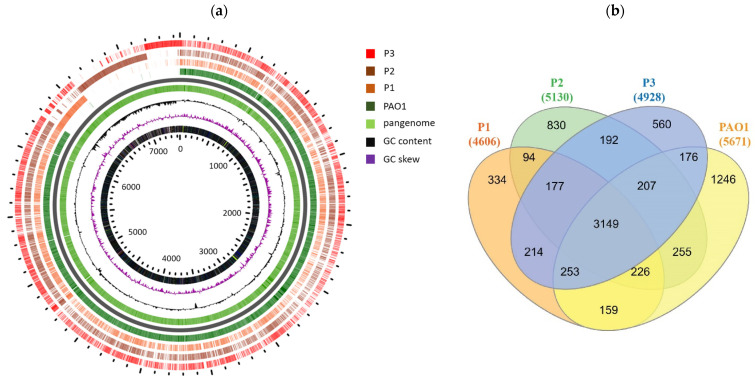
Whole genome sequencing (WGS) of the persister isolates. (**a**) Genomic comparison of the PAO1 and studied isolates. Using gView Server, the pan genome of the four isolates was constructed and the genes were mapped against it. (**b**) Venn diagram of genes in the three persister *P. aeruginosa* isolates and PAO1 strain. The four strains shared in 3149 genes, while 334, 830, and 560 genes are unique to P1, P2, and P3, respectively.

The circular map representing the entire genome assembly is shown in Figure 4. The circular image depicts the comparison of the genome of isolates P1, P2, and P3 to the reference genome of the PAO1 (NC_002516) strain. From the inner to outer circle is depicted the nucleotide position, GC content, GC skew (of the PAO1), and genes of isolates P1, P2, and P3, respectively. In addition, a comparison of the PAO1 genome with persister genomes revealed the presence of a number of genes (2401) in the persister genomes that were absent in the PAO1 genome (Supplementary Table S3).

### 3.5. Antimicrobial Resistance Elements

**Figure 5:**
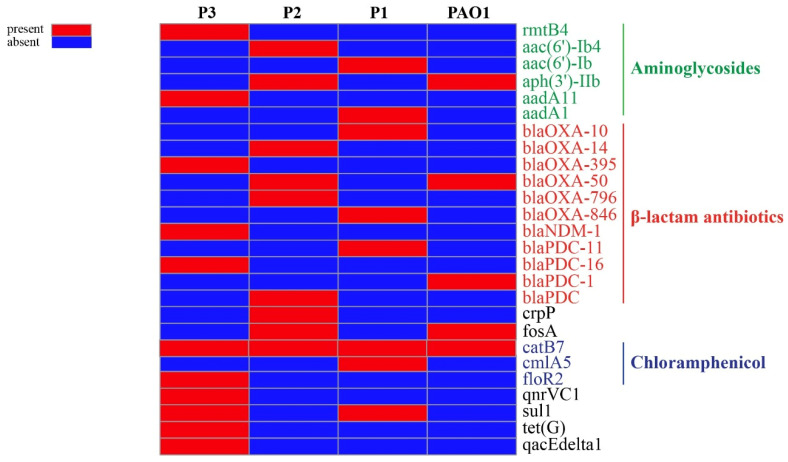
Resistome profiling of antimicrobial modifying enzymes in persister isolates compared to PAO1 standard strain. Antibiotic resistance gene profiling was carried out using NCBI’s AMRfinder as well as ‘resistance gene identifier’ v5.05.5 against the CARD database. Heatmap was constructed using Clustvis online tool.

Resistome profiling was carried out to detect the resistance elements contributing to the MDR-pattern of these persisters forming isolates against anti-pseudomonas antibiotics. The analysis of antibiotics and antiseptic resistance elements pinpointed 26 resistance genes distributed among the isolates, 21 of them only exist in these persister isolates and 4 are shared with the PAO1 genome, while the blaPDC-1 gene is present in only the standard strain of PAO1 (Figure 5). These resistance elements represent a cocktail of resistance elements against most of the clinically used antibiotics, including the major aminoglycosides modifying enzymes acetyltransferases: *aac(6′)-Ib* and *aac(6′)-Ib4,* phosphotransferase: *aph(3′)-IIb,* nucleotidyltransferases: *aadA1* and *aadA11* beside the 16S-rRNA methylase *rmtB4*. Variants and sub-variants of different classes of β-lactamases were detected, including *bla_OXA-10_*, *bla_OXA-14_*, *bla _OXA-395_*, *bla _OXA-50_*, *bla _OXA-796_*, and *bla _OXA-864_*. Several pseudomonas-derived cephalosporinase (PDC) variants were also detected, i.e., bla *_PDC_*, bla*_PDC-1_*, bla*_PDC-11_*, and bla*_PDC-16_* and finally the *bla_NDM-1_* gene, which encodes for the New Delhi metallo-β-lactamase 1. Acquired resistance elements against chloramphenicol were also detected, including *catB7, cmlA5,* and *floR2*, while individual resistance factors against tetracycline (*tetG*), fosfomycin (*fosA*), sulphonamides (*sul1*), novel ciprofloxacin-modifying enzyme encoding gene *crpP*, quinolone resistance determinant (*qnrVC1*), and the antiseptic resistance gene (*qacEΔ1*) are mainly found in isolate number 3. The broad collection of antibiotic resistance genes found in the genomes are the reason for the MDR of these isolates.

Aside from the antibiotic modifying enzymes, the persisters’ genome harbored gene clusters that correspond to the efflux pump-mediated resistance. Genome analysis identified 24–30 genes shared among the three isolates. These genes code for membrane fusion proteins (MFP), resistance nodulation division proteins (RND), and outer membrane proteins (OMP) that constitute efflux pump systems (Table 3). Based on the identified gene clusters, nine multidrug resistance efflux pumps (MexAB-OprM, MexCD-OprJ, MexEF-OprN, MexGHI-OpmD, MexJK-OpmH, MexMN-OprM, MexPQ-OpmE, MexXY-OprM, and MexVW-OprM) were chromosomally encoded in the persister isolates. Moreover, mutagenic analysis of the MexAB-OprM efflux pump regulators recognized a few point mutations in the MexAB-OprM repressors (MexR, NalD, and NalC).

### 3.6. Global Phylogenetic Analysis of the Persister Isolates

**Figure 6:**
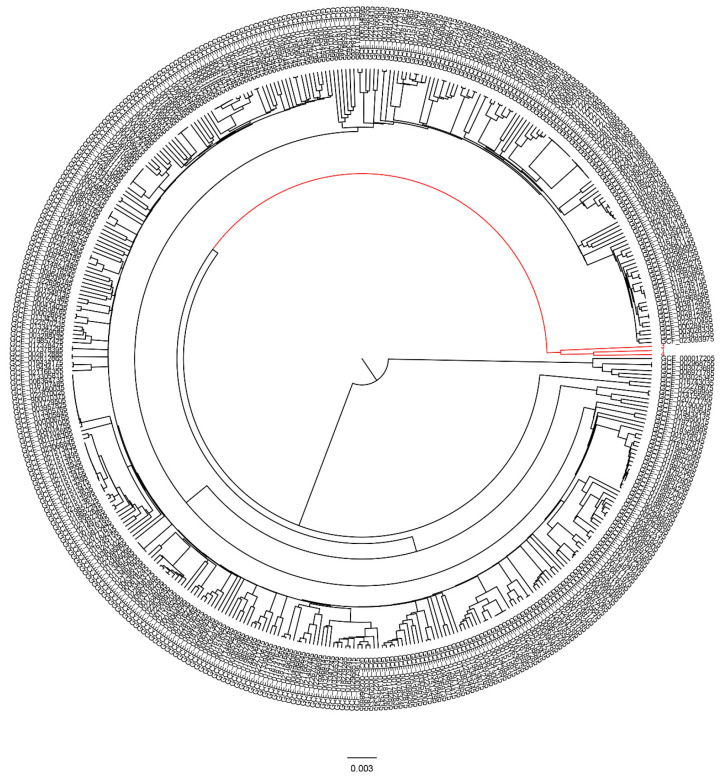
Phylogenetic tree analysis of persister isolates compared to the genome of 445 *P. aeruginosa* isolates downloaded from the NCBI database. The phylogenetic tree was constructed based on the MASH distance using Ondov et al. [35]. Our persister isolates were placed in a unified but separate clade (indicated by the red color).

The phylogenomic location of the studied isolates was performed. Completely closed genomes of 445 *P. aeruginosa* isolates were downloaded from the public database and compared with these persister-forming isolates. When phylogenomically compared to previously known *P. aeruginosa* strains from different countries, the studied isolates were placed in a unified but separate clade (Figure 6 and Supplementary Table S4).

## 4. Discussion

Chronic infections represent a terrible therapeutic challenge and have a puzzling property. These infections are difficult and even impossible to eradicate. There is growing evidence that bacterial persisters are involved in the relapse of bacterial infections [42]. Th characterization of persister cells is important, not only in regarding the understanding of population dynamics but also concerning antibiotic tolerance in chronic-infections therapy [43]. Few studies have assayed persister formation in clinical isolates from complex host environments [44,45]. In the current study, the characterization of *P. aeruginosa* persisters isolated from chronic respiratory tract infections was performed using phenotypic and genotypic methods.

Initially, we isolated *P. aeruginosa* multidrug-tolerant persister forming strains. Although 100-fold MIC is unlikely to be achieved in the therapy of clinical infections, the application of high doses of antimicrobials would select for high persister mutants. The treatment with 100-fold MIC is a method of choice to identify highly drug-tolerant persister cells of *P. aeruginosa* [46]. Accordingly, in our study, three different *P. aeruginosa* isolates were identified as persister-forming strains. These isolates were recovered from 38 *P. aeruginosa* clinical isolates (8% persister-forming capacity). A low persister level, approaching 1% of the total population, was reported by Mulcahy et al. [44]. While a higher persister forming percentage (19%) was reported recently [45], their isolates were only obtained from CF infections, which is a deadly disease accompanied with a chronic respiratory infection. 

Two of the three persister isolates (P2 and P3) formed both large colonies and small colonies on agar plates. Similarly, it was reported that *S. aureus* persisters induce a phenotypic change into small colony variants due to genomic inversion [47]. Furthermore, a recent study indicated that *P. aeruginosa* can persist for a long time in an acidic environment, through phenotypic alteration to a slow-growing colony phenotype [13].

The frequency of persisters was calculated for each isolate, and it ranged from 0.0017 to 0.0027 (approximately 10^−3^, meaning 1 out of 10^3^ cells can form persister). Our result is quite consistent with the results of Salcedo-Sora and Kell [48], who reported a persister level within the range of 0.0012–0.0016. However, our results reveal much higher frequencies than the 10^−5^ and the 10^–6^ reported previously [24,49].

*P. aeruginosa* strains are intrinsically resistant to a variety of antimicrobials, and can further develop resistance during therapy [3]. In this study, the MIC results revealed that the persister-forming isolates were highly resistant to most of the tested antibiotics. Similarly, a recent study of *Pseudomonas* CF isolates revealed that more than 75% of the CF isolates were resistant to at least half of the antibiotics. Additionally, 25% of their isolates were found to be resistant to all the tested antibiotics, and only 36% of CF-isolates were resistant to tobramycin [50]. Furthermore, it was reported that persister (Hip) isolates were resistant to ciprofloxacin by 72.4% [45]. In this investigation, the time-killing curves using colistin, meropenem, and tobramycin revealed that the curves substantially differentiate not only between clinical isolates of *P. aeruginosa* but also between the different antibiotics. For example, isolate P1 showed a higher number of survival after exposure to tobramycin and meropenem, while isolate P2 had more survivor cells upon exposure to colistin.

The threat of *P. aeruginosa* is complicated due to its ability to form biofilms that provide a protected environment to bacterial cells which help in tolerating various stresses, including antimicrobials [51,52]. It was reported that the formation of *P. aeruginosa* persisters and biofilms are interrelated [53]. The biofilm formation was evaluated in the three persister-forming isolates, in addition to the PAO1 standard strain and a non-persister clinical isolate. All the tested isolates were strong biofilm formers; however, the persister isolates had stronger biofilms (as their OD was twice that of the PAO1 strain). In contrast, a previous study reported that the biofilm of PAO1 and their persister strain had the same strength [26].

Genomics has been a key element in the study of *P. aeruginosa* evolution. It has been reported that *P. aeruginosa* strains had a variable genome size of approximately 5–7 Mbp [54]. The omnipresence of *P. aeruginosa* may be related to its high genome plasticity. The genome of the *P. aeruginosa* PAO1 standard strain was sequenced approximately two decades ago [54]. However, the genetic mediators of persister formation for *P. aeruginosa* are poorly understood [4]. In the current study, genome sequencing of persister-forming isolates revealed that they had smaller genomes (4.66–4.9 Mbp), compared to PAO1-genome (6.3 Mbp) sequenced previously [55], and the genome of *Pseudomonas* CF-isolates (6.36 Mbp) recently sequenced [50]. The smaller genome size for our isolates could be due to the short-reads-based sequencing and assembly used in our study. Furthermore, it is documented that *P. aeruginosa* can customize its genome as individual strains have the ability to acquire or discard genomic segments to fit their needs for survival in virtually any environment [56].

Resistome profiling indicated the presence of a broad collection of antibiotic-resistance genes in our persisters’ genome, with 25 resistance genes distributed in persister-isolates, most of which confer resistance to β-lactams (11 genes), aminoglycoside resistance (6 genes), and chloramphenicol resistance (3 genes), while resistance to other antibiotic classes is mediated by one gene each. CARD analysis revealed that the CF-isolates genome contains a high number of resistance genes, including beta-lactam resistance genes (*n* = 6), efflux pumps (*n* = 37), antibiotic inactivation enzymes (*n* = 8), and only one aminoglycoside resistance gene [50].

Multidrug efflux pumps constitute a group of antibiotic resistance elements that are a core part of bacterial genomes [57]. The *P. aeruginosa* PAO1 genome contains several drug efflux systems, predominantly of the RND and MFS families [56]. In the current study, several multidrug efflux tripartite pumps that encoded in operon were recognized at the genome level in our persister isolates (6–8 classes of the RND multidrug efflux pump-operon in each genome), which explains their profound resistance against tested antibiotics. Consistent with our results, MexA/B pumps were reported to be closely associated with antimicrobial resistance and biofilm formation in clinical *P. aeruginosa* isolates recovered from respiratory infections [58]. 

It was reported previously that mutations in mexR, nalC, and nalD regulatory genes yield a MDR phenotype in *P. aeruginosa* [59,60]. Surprisingly, the identified point mutations within the MexAB-OprM efflux pump regulatory genes (MexR, NalC) have been previously described in clinical MDR–*P. aeruginosa* isolates [61,62]. In addition, an inactivating mutation in *mexZ*, encoding a repressor of the MexXY-OprM pump, was reported in *P. aeruginosa* persister isolates from CF patients [44]. All of these mutations favored the overexpression of these pump systems and caused increased resistance to different antimicrobials. Future work will focus on studying the transcriptional changes associated with persister formation to identify potential targets for the anti-persister drug.

Our results in resistome profiling of persister isolates allow the identification of genes that are likely to be involved in the antibiotic resistance of *P. aeruginosa* persister isolates. Knowledge of the complete genome sequence and encoded processes provides a wealth of information for the discovery of new antibiotic targets and for the development of more effective strategies to treat the life-threatening opportunistic infections caused by *P. aeruginosa* in humans.

Furthermore, phylogenetic analysis indicated that persister isolates belong to a unified but separate clade rather than the deposited *P. aeruginosa* strains in the public repositories. Further investigation is required to explore the validity and features of this clade. Similarly, the phylogenetic analysis of *P. aeruginosa* CF isolates performed by Datar et al. [50] revealed their unique clustering and indicate their location in different clades. To the best of our knowledge, our study is the first to study the genome sequence of persister isolates from local hospitals in Egypt.

## 5. Conclusions

The present study characterized, for the first time in Egypt, *P*. *aeruginosa* persister isolates. The persister isolates were strong biofilm producers and have a highly resistant phenotype when compared to the PAO1 standard strain. These results were confirmed by genome sequencing revealing a large number of resistance genes. Furthermore, WGS revealed a smaller genome that belong to a unified but separate clade compared to the genome of other *P*. *aeruginosa* strains deposited in the GenBank.

## Figures and Tables

**Table 1:** Resistance profiles of the recovered persister strains and PAO1 standard strain.

Strain Code	MICs (µg/mL)								MDR
	CHL	AK	LEV	CEP	FEP	MEM	CT	TOB	
PAO1	2 (S)	4 (S)	0.5 (S)	4 (S)	1 (S)	0.5 (S)	1 (S)	2 (S)	-
P1	256 (R)	128 (R)	8 (R)	256 (R)	64 (R)	64 (R)	16 (R)	128 (R)	5
P2	512 (R)	512 (R)	8 (R)	512 (R)	128 (R)	16 (R)	32 (R)	128 (R)	5
P3	256 (R)	128 (R)	16 (R)	512 (R)	64 (R)	16 (R)	16 (R)	512 (R)	5

**S:** Sensitive, **R:** Resistant, **MDR:** Multidrug Resistant, **AK:** Amikacin, **CEP:** Cefoperazone, **FEP:** Cefepime, **MEM:** Meropenem, **CT:** Colistin, **TOB:** Tobramycin, **LEV:** Levofloxacin, **CHL:** Chloramphenicol.

**Table 2:** Biofilm forming capacity of persister strains compared to PAO1.

Isolate	Absorbance (OD) at 570 nm	Biofilm Forming Capacity
P1	0.967	S
P2	0.831	S
P3	0.872	S
PAO1	0.584	S
NP	0.547	S
ODc	0.1	Control (no biofilm)

**S**: strong biofilm forming, **NP:** non persister isolate.

**Table 3:** Genotypic characteristics of efflux pump systems in *P*. *aeruginosa* persister isolates.

Persister Number	Efflux Pump Component			Efflux Pump	Mutation in MexAB-OprM Efflux Pump Regulatory Proteins
	MFP	RND	OMP		
P1	MexA	MexB	OprM	MexAB-OprM	MexR (V126E), NalC (S209R, G71E)
	MexC	MexD	OprJ	MexCD-OprJ	
	MexJ	MexK	OpmH	MexJK-OpmH	
	MexM	MexN	OprM	MexMN-OprM	
	MexP	MexQ	-		
	MexV	MexW	OprM	MexVW-OprM	
P2	MexA	MexB	OprM	MexAB-OprM	MexR (V126E), NalC (S209R, G71E)
	MexC	MexD	OprJ	MexCD-OprJ	
	MexE	MexF	OprN	MexEF-OprN	
	MexG	MexH,I	-		
	MexJ MexM	MexK MexN	OpmH OprM	MexJK-OpmH MexMN-OprM	
	MexP	MexQ	-		
	MexV	MexW	OprM	MexVW-OprM	
P3	MexA	MexB	OprM	MexAB-OprM	MexR (V126E), NalC (S209R, G71E)
	MexC	MexD	OprJ	MexCD-OprJ	
	MexE	MexF	OprN	MexEF-OprN	
	MexG	MexH,I	OpmD	MexGHI-OpmD	
	MexJ	MexK	OpmH	MexJK-OpmH	
	MexM	MexN	OprM	MexMN-OprM	
	MexP	MexQ	-		
	MexV	MexW	OprM	MexVW-OprM	

## Data Availability

Not applicable.

## Supplementary Materials

The following supporting information can be downloaded at: https://www.mdpi.com/article/10.3390/pathogens12030426/s1, Table S1: The batch number of antimicrobial agents used in the current study; Table S2: CLSI breakpoints of antimicrobial agents used in the current study; Table S3: The list of genes that found in persister strains genome and absent from PAO1 genome; Table S4: List of the 445 *P. aeruginosa* isolates downloaded from the NCBI database and used for Phylogenetic tree analysis
