# Supplementary material for: Genome Analysis of Pseudomonas aeruginosa Strains from Chronically Infected Patients with High Levels of Persister Formation
Source: Pathogens. 2023 Mar 8;12(3):426. doi: 10.3390/pathogens12030426 (PMC10051920; doi:10.3390/pathogens12030426)
Supplement: Supplementary file 1 [file pathogens-12-00426-s001.zip › Supplementary Table S1.pdf]

Supplementary Table S1: The batch number of antimicrobial agents used in the current study

Antimicrobial agent	Batch number
Amikacin	227184
Cefoperazone	01714322
Cefepime	04713622
Chloramphenicol	01200424
Colistin	NB2 109-07B
Levofloxacin	213663
Meropenem	027601221
Tobramycin	2209292
