# Supplementary material for: Genome Analysis of Pseudomonas aeruginosa Strains from Chronically Infected Patients with High Levels of Persister Formation
Source: Pathogens. 2023 Mar 8;12(3):426. doi: 10.3390/pathogens12030426 (PMC10051920; doi:10.3390/pathogens12030426)
Supplement: Supplementary file 1 [file pathogens-12-00426-s001.zip › Supplementary Table S2.pdf]

Supplementary Table S2: CLSI breakpoints of antimicrobial agents used in the current study

Antimicrobial agent	S	I	R
Chloramphenicol	≤ 2	4	≥ 8
Amikacin	≤ 16	32	≥ 64
Levofloxacin	≤ 2	4	≥ 8
Cefoperazone	≤ 16	32-64	≥ 128
Cefepime	≤ 8	16	≥ 32
Meropenem	≤ 2	4	≥ 8
Colistin	≤ 2	4	≥ 8
Tobramycin	≤ 4	8	≥ 16

S: sensitive, **I: intermediate,** **R: resistant**
